# Control of Laser Induced Cumulative Stress for Efficient Processing of Fused Silica

**DOI:** 10.1038/s41598-020-60828-3

**Published:** 2020-03-02

**Authors:** Qi Sun, Timothy Lee, Martynas Beresna, Gilberto Brambilla

**Affiliations:** Optoelectronics Research Centre, University of Southampton, Southampton, SO17 1BJ United Kingdom

**Keywords:** Laser material processing, Optoelectronic devices and components

## Abstract

Laser irradiation of silica glass is shown to trigger redistribution of material resulting in accumulation of stress and refractive index modification, and the rearrangement of the glass network has a significant impact on the quality of laser written optical components. We propose an alternative laser writing approach for achieving the desired refractive index and optical phase profiles through improved material stress control, demonstrated using both Gaussian and Bessel writing beams. The new material processing strategy is successfully adapted for implementing photonic circuits and diffractive elements with greater efficiency due to improved uniformity and symmetry of the induced index modification.

## Introduction

In recent years, femtosecond laser writing has become a key technology for processing transparent materials. Due to the nonlinear nature of absorption, the femtosecond laser-induced change is confined to material at the focal volume, which enables inscription of 3D structures including waveguides^1^, couplers^2^, spatially variant waveplates^3^ and diffractive optical elements (DOEs)^4^. Also, femtosecond laser writing has been applied in data storage^5^ and selective etching^6,7^. In each case, laser radiation initiates a redistribution of the glass network leading to stress. The stress leads to a partial change in the refractive index^8^, a decrease in the resistance to chemical corrosion and weakened mechanical performance of the material^9^. In fused silica, depending on laser parameters, there are 3 types of modifications: continuous modification, nanostructure formation and nano-void formation^10^. With different laser energy deposition levels, these modifications can induce either tensile or compressive stress distribution^11^ around the laser affected zone which will then result in a positive or negative change of refractive index. For many applications, the refractive index change Δ*n* is the most important parameter; thus a significant effort was dedicated to achieving the highest value whilst neglecting the importance of uniformity. Previous papers have demonstrated that the index change can be controlled by energy deposition density and pulse duration^12^. However, because of the stress relaxation mechanism, at a high energy density level, the strong stress inside the material will lead to the formation of cracks^13^. For fused silica, the highest Δ*n* induced by a femtosecond laser reached 2.2 × 10^−2^, which was achieved at tight focusing conditions (an oil immersion lens with NA = 1.25) and at a short writing-beam wavelength of 522 nm^14^. However, many applications require modification of large material volumes or writing high aspect-ratio structures with the desired refractive index modulation, which is challenging but essential. Such examples include glass cutting, inscription of micro-fluidic channels, photonic circuits, and DOEs. For example, the efficiency of DOEs relies on optical phase change, which is not only affected by Δ*n* but also physical thickness of the phase retarder. Thus, sufficient phase change can be achieved only with a thick layer of the modified material, further increasing the importance of homogeneous modification. Previous research indicates that writing with a Bessel beam could serve as a viable solution for this challenge^15^. Compared to a Gaussian beam, a Bessel beam has a much longer focal depth with more uniform intensity distribution as a result of its non-diffractive nature^16^. However, during the inscription of tall structures, the stress and refractive index modification of previously irradiated regions can distort the writing beam, which impairs the index and stress uniformity of the overall structure.

In this paper, we demonstrate that the refractive index distribution is affected by accumulated stress and investigate a new approach for controlling the stress distribution during laser induced modification of transparent materials. Typically, the material is modified by scanning the writing beam in a raster line pattern known as ’multiscan’^17^, which leads to an asymmetric stress distribution and even crack formation. However, by applying different scanning techniques we are able to control the refractive index distribution and reduce the negative impact of the stress. The method is effectively used for imprinting single- and multi-layered structures. We then demonstrate how optimised stress distribution improves performance of DOEs and photonic circuits. More generally, the method could also be applied for controlling etching profiles, implementing optical waveguides with gradient refractive index distribution or more complex DOEs.

## Results

**Figure 1:**
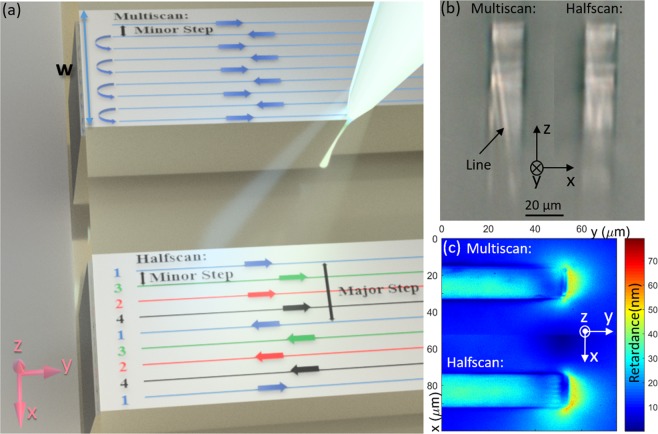
(**a**) Schematic of multiscan (top) and halfscan (bottom) laser writing methods. Arrow shows the laser moving direction. For the halfscan schematic, colours denote different writing order (blue-red-green-black). (**b**) Microscope image of Bessel beam written waveguide cross sections with multiscan (left) and halfscan (right) methods. Both waveguides have a minor step of 0.2 *μ*m and the halfscanned waveguide has a major step of 1.6 *μ*m. Both waveguides have widths (in *x* direction) of 20 *μ*m and are written with a laser pulse energy of 0.65 *μ*J and pulse density of 10^5^ pulses/mm. (**c**) Top view (-*z* direction) retardance measurement of waveguides in (**b**).

 Figure 1a compares the schematic of femtosecond laser written waveguides with multiscan and halfscan writing methods. The width *w* (in the *x* direction) of each waveguide is 20 *μ*m. For the multiscan method, the laser scanlines are written consecutively one next to the other with a minor step of 200 nm in the *x* direction. As a result, each scanline will be affected by the stress distribution of the previous line, which results in the stress accumulation. The left side of the microscope image in Fig. 1b shows a traditionally written (i.e. multiscanned) waveguide cross section. The brighter area shows the higher laser induced index change and thus stronger guidance of microscope illumination light. The uneven index distribution in the *z* direction is a result of uneven axial intensity distribution of Bessel beam itself^18^. Due to the stress accumulation, the multiscanned waveguide forms a gradient index change which results in a visible index cross line. This line forms and can be easily observed by microscope when the laser pulse energy is high, approximately 0.65 *μ*J for our Bessel beam writing set up. However, even when the index cross line is not obvious for lower powers, the asymmetric stress distribution still exists with the conventional multiscan technique. These features remain present in the cross section after polishing, which also tends to introduce large cracks due to the high stress. The inhomogeneous stress distribution produces an uncontrollable index profile thus reducing the efficiency of any diffractive optics based on such structures.

To reduce the stress asymmetry, we use a new ‘halfscan’ method depicted in the bottom waveguide in Fig. 1a, where the line order is rearranged. Different colours denote the line writing order (blue-red-green-black). The main principle is to keep each scanline separated from the previously written scanline by a major step (8 times larger than the minor step) and finally achieve an overall minor step structure. As each subsequent scanline has a larger gap than the multiscan method, this reduces the effect of the previous scanline on the writing of the next line. Also, the larger scan step reduces the chances of damage. The right waveguide in Fig. 1 shows the improved profile obtained with 1.6 *μ*m major step halfscan writing method, compared to the multiscan written waveguide which shows an obvious index contrast line.

In order to investigate how the stress was distributed with both laser scan methods, we then performed top view retardance measurements of the waveguides (Fig. 1c). Note that for these test waveguides, only one end reaches the sample end face (to allow cross section imaging), and the other end terminates within the silica. Indeed, for our analysis we focus on the portion away from the extremities, since the anisotropy around the ends is not representative of the rest of the structure. The halfscanned waveguide shows a more even retardance profile across the waveguide’s width in the *x* direction compared to the multiscanned waveguide. The retardance distribution is related to stress profile of structure, which will be analysed in discussion section.

**Figure 2:**
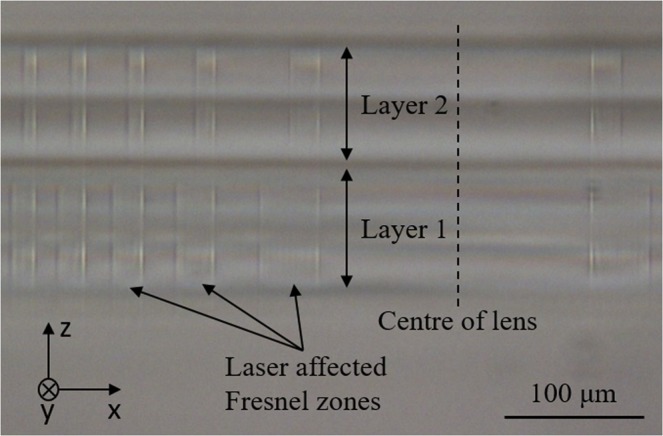
Cross section of part of a 2-layer Fresnel lens written with a Bessel beam using halfscan technique at power of 90 mW. Diameter of Fresnel lens is 2 mm and thickness of each layer is 100 *μ*m. The dotted line is the centre of the Fresnel lens.

To demonstrate that this technique can be applied to more complex multilayer structures, we wrote 50/50 diffractive gratings and 2-layer Fresnel lenses. Figure 2 shows the microscope image of a two layer Fresnel lens cross section, showing the region near the centre (the dotted line shows the Fresnel lens centre). The Fresnel zones with increased refractive index are clearly visible, and decrease in width with radial distance (in the  −*x* direction) from the centre. The Fresnel lens was written with a Bessel beam using the halfscan technique at a power of 90 mW. The thickness of each layer (in *z* the direction) is 100 *μ*m. Some of the laser affected Fresnel zones are marked in the image. Notice that the laser affected zone appears quite smooth compared to left multiscanned waveguide in 1b. Table 1 shows the diffractive efficiency results at a measurement wavelength of 633 nm. For the halfscanned grating, the first order diffraction efficiency of grating improved by 36% (from *η* = 17.7% to 24.1%) compared to the multiscan method. With the halfscan method writing, the Fresnel lens reached a focal efficiency of 55%, with a theoretical limit of 65%.

**Table 1:** Efficiency of diffractive optical elements inscribed with different writing methods.

	50/50 Grating	2-layer Fresnel Lens
Multiscan	17.7%	—
Halfscan	24.1%	55%

**Figure 3:**
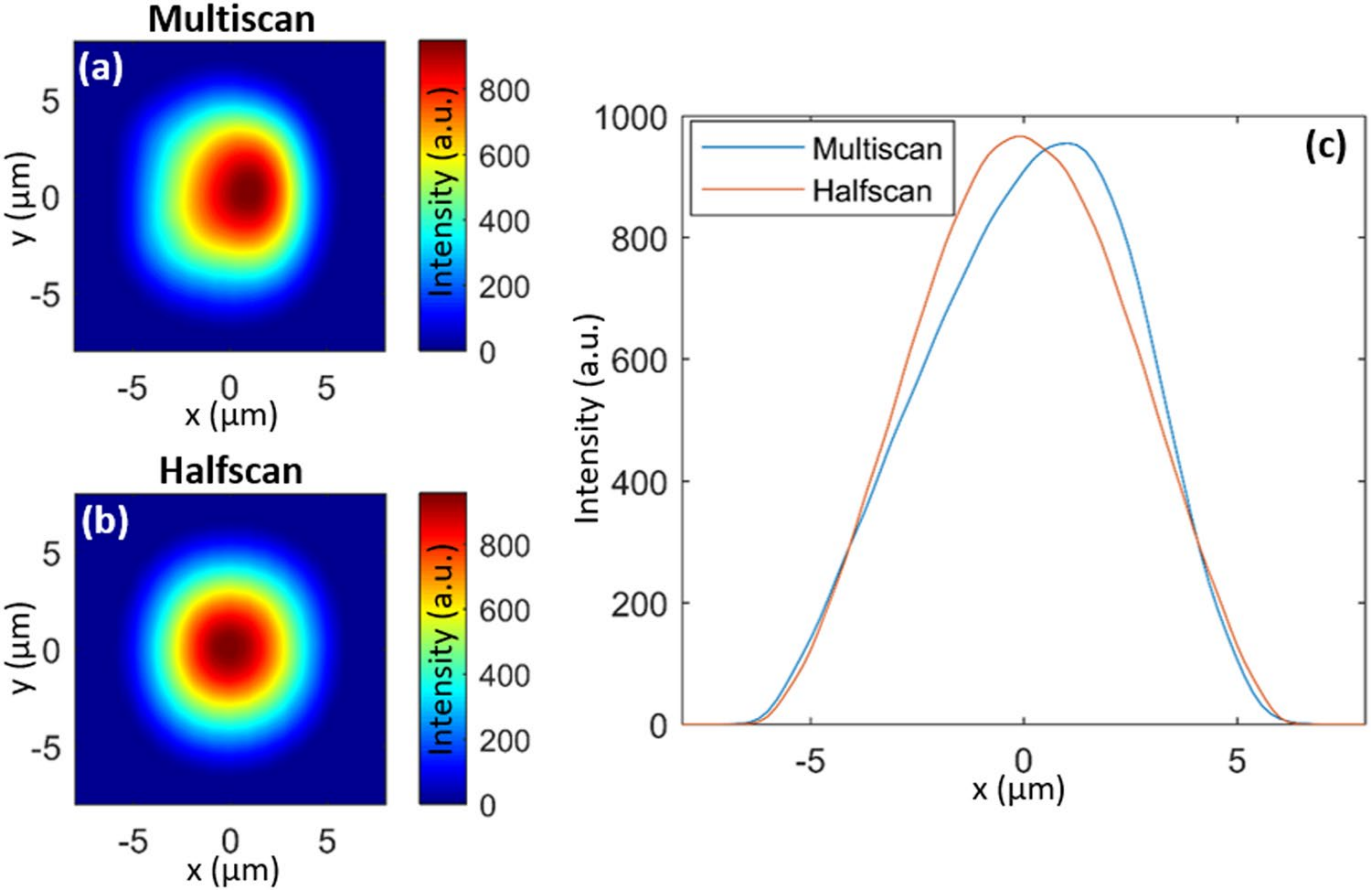
The output mode intensity profile of waveguides written by (**a**) multiscan and (**b**) halfscan. (**c**) Central cut-line along *y* = 0 of intensity profiles. The *x* axis is centred on the middle of the 10 *μ*m width waveguide.

The observed effects are not limited to Bessel beam writing, as we also achieved a more symmetric structure with the halfscan technique whilst writing low-aspect-ratio waveguides (10 × 10 *μ*m cross section) with a Gaussian beam. Figure 3a,b show the output modes from waveguides inscribed using multiscan and halfscan methods, respectively. The mode profile was captured by an IR camera (MicronViewer 7290A, Electrophysics). These waveguides were written with a 1030 nm Gaussian beam with a pulse energy of approximately 0.3 *μ*J and pulse density of 5 × 10^3^ pulses/mm along each scanning line. According to the mode intensity plot comparison in Fig. 3c, the waveguide written with the halfscan method has a more symmetric mode profile than that of the multiscan method.

## Discussion

**Figure 4:**
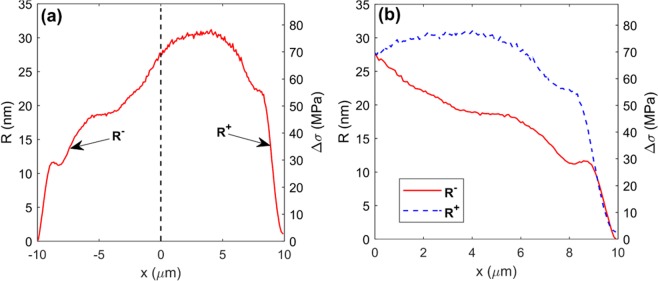
(**a**) Retardance *R* and principle stress difference Δ*σ* distribution across waveguide, of width *w* = 20 *μ*m, written with multiscan method at a laser power of 130 mW. *x* axis shows the distance to the waveguide centre (half width point). (**b**) Flipped image of (**a**) along waveguide centre point (i.e. along *x* = 0 in (**a**)) where red solid *R*^+^ curve and blue dashed *R*^−^ curve indicate left and right sides respectively.

**Figure 5:**
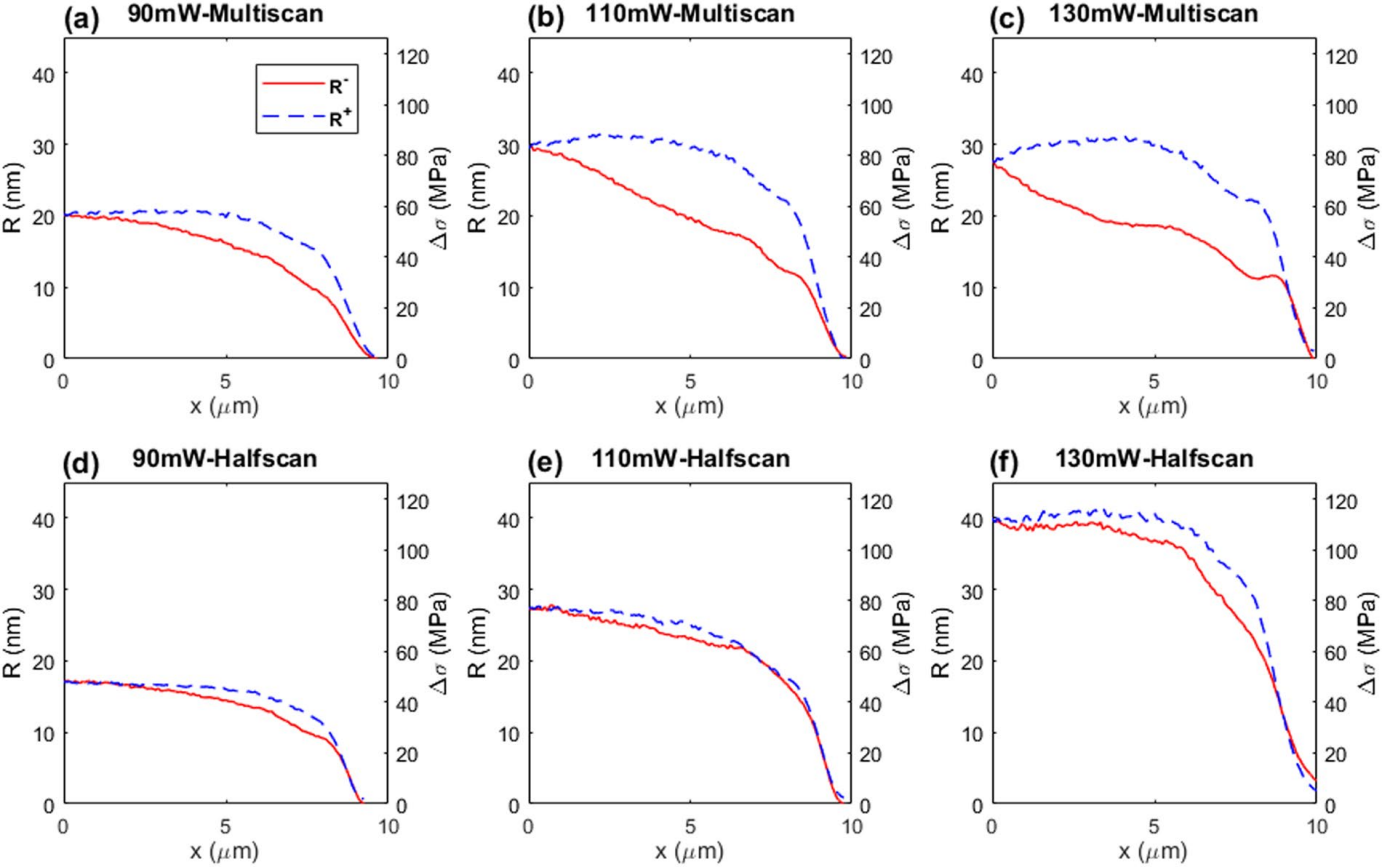
Flipped waveguide stress distributions for different power level and scanning method.

For the structure written with the Bessel beam, from Fig. 1c, with the retardance distribution *R* across waveguide width *w*, we calculated the principle stress difference^19^  Δ*σ* by Eq. 1 (see right axis in Fig. 4a):

1 $$\Delta \sigma =\frac{R}{C\cdot T}$$

where *C* = 3.55 × 10^−12^ Pa^−1^ is the photoelastic coefficient between two principal stresses in silica, and *T* = 100 *μ*m is the thickness of waveguide in *z* direction. Flipping the left side of the waveguide stress distribution curve along *x* = 0 in the Fig. 4a produces Fig. 4b, which is more convenient for comparing and analysing the symmetry of the waveguide profiles. Figure 5 shows the flipped waveguide stress profiles at 3 laser writing powers (90, 110 and 130 mW, corresponding to pulse energies of 0.45, 0.55 and 0.65 *μ*J, respectively) for both multiscan and halfscan waveguides.

With both scanning methods, the peak stress level increases with increasing writing power. However, for the 130 mW multiscan written waveguide (Fig. 5c), the stress maintains the same level with that written at 110 mW. This is due to the stress accumulation in one direction and finally forming a nano-crack inside the waveguide at the higher power. These nano-cracks then release part of the stress inside and lower the overall stress level^20^. For the halfscan writing method, the evenly distributed stress across the waveguide enables a higher tolerance of laser writing power thus higher achieved overall stress level. For different waveguide widths, further waveguides with a similarly symmetric profile have been written with the halfscan method to confirm that the results are indeed reproducible. For laser written structures, especially DOEs, higher writing power normally leads to higher laser induced index change. With the halfscan method, it is easier to achieve the same phase change in a shorter device depth, which is beneficial for compact device fabrication and saving time. For a quantitative comparison, we calculated the average mean square error (AMSE) of the *R*^+^ and *R*^−^ curves in each figure in Fig. 5 with Eqs. 2 and 3.

2 $$\bar{R}(x)=\frac{1}{2}({R}^{+}(x)+{R}^{-}(x))$$

3 $$AMSE=\frac{1}{w}{\int }_{0}^{w/2}{[{R}^{+}(x)-\bar{R}(x)]}^{2}+{[{R}^{-}(x)-\bar{R}(x)]}^{2}dx$$

Table 2 shows the AMSE results. The AMSE of multiscanned waveguides rapidly increases from 2.75 nm^2^ to 19.85 nm^2^ with increasing writing power from 90 mW to 130 mW, which shows a badly degraded asymmetric stress profile. For the halfscan method, the AMSE maintains a low level (2.12 nm^2^) even with a high power writing (130 mW).

**Table 2:** AMSEs of waveguide stress symmetry.

	90 mW	110 mW	130 mW
Multiscan	2.75 nm^2^	14.02 nm^2^	19.85 nm^2^
Halfscan	0.48 nm^2^	0.32 nm^2^	2.12 nm^2^

The maintenance of symmetry and stress uniformity is beneficial for more complex structure fabrication. Indeed, the 50/50 grating shows a 36% efficiency improvement by using halfscan over multiscan. For the Fresnel lens, the efficiency is high and approaches the theoretical maximum limit; the discrepancy being due to the theoretical value assuming an infinitely thin device with ideal step-transitions in profile phase, neither of which can be perfectly realised in practice. Finally, it is worth noting that the performance of the DOEs could be further enhanced by varying the pulse density of the scanlines while writing, so as to provide a continuously varying phase profile (rather than a discrete single or two level phase profile) which allows a higher theoretical efficiency limit. The halfscan method would be especially advantageous in this instance as very precise control of the phase profile is essential, and multiscan writing would introduce too much distortion.

**Figure 6:**
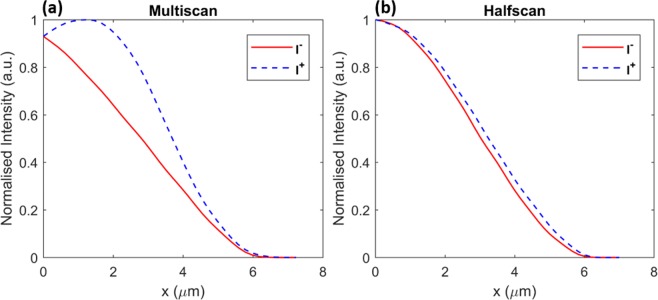
Flipped normalised mode intensity distributions for waveguides written by (**a**) multiscan and (**b**) halfscan.

As for the waveguides inscribed with the Gaussian beam, we also calculated the AMSE of their mode intensity profiles to compare the modal symmetry obtained by the multiscan and halfscan approaches. Figure 6 shows the comparison of flipped images of the central mode intensity cut-lines. To eliminate the error from different peak intensities, we normalised the peak intensity to unity. The AMSEs of the halfscanned and multiscanned waveguide mode intensities are 2.5 × 10^−4^ and 7.3 × 10^−3^ respectively. The halfscanned waveguide therefore achieves a much more symmetric mode profile than the multiscanned waveguide (30 times more symmetric in terms of AMSE).

Generally, index symmetry and index uniformity are two distinct properties and their importance (or lack thereof) is application-dependant. As an example, it is worth noting that for some applications like waveguides bends or gradient gratings, an asymmetric index distribution could be in fact preferential. Meanwhile, a straight waveguide might benefit from index profile symmetry, but not necessarily transverse uniformity (both graded and top-hat profiles have their benefits, for example).

For different index change based femtosecond laser written applications, we thus propose five different scan methods: multiscan, inward scanning, outward scanning, segmented multiscan, and halfscan. Each method has its advantages and disadvantages. While the multiscan method is the simplest and quickest among all methods, it leads to the strongest asymmetry of the refractive index and stress. The inward scanning method scans from outside edges of structure to the central line. In contrast, the outward scanning method scans from the central line to outside edges. Both inward and outward methods produce a symmetric profile with different refractive index distribution. For waveguides writing, outward scanning gives a smaller mode field diameter than inward scanning because the stress in the centre of the waveguide is higher. However, while symmetric, the index profile is not even (because it is essentially similar to two multiscans joined back-to-back), which is unsuitable for devices requiring uniform profile such as diffractive optics. The multiscan, inward, and outward scan techniques benefit from their simplicity, being quick to code for prototype testing.

The more complex segmented multiscan approach divides the structures into several parts, and scans in a multiscan way for each part, with the scanning order cycling through each part. For Gaussian beam writing waveguides, the modification is fairly symmetric and also uniform. However, with Bessel beam writing for high aspect ratio structures, it does not give sufficient uniform index modification, because after the first set of major-separation scan-lines are written, the second set is written next to it, separated only by minor separation. Finally, the halfscan can be considered an improved version of the segmented multiscan and works well for Gaussian and Bessel beams, i.e. for both low and high aspect ratio modifications. It gives both symmetric modification as well as much improved uniformity, especially with high power writing.

Apart from DOEs and waveguides, our stress controlling method could be further extended to laser machining and large waveguide fabrication, where uniform stress control is essential but challenging. Moreover, as a result of laser induced localized densification, the glass network is also rearranged by the surrounding stress in the laser affected zone^21^. With higher compressive stress, the Si-O-Si bond angle of silica decreases^22^ and the size of the SiO_4_ ring is also reduced to a lower number^23^. For laser assisting etching, higher index change^23^ and lower number SiO_4_ rings^24^ tend to give a faster etching rate, so our stress control method could also be applied to implement a more selective and faster fs laser assisting etching process.

## Methods

The experiments were carried out with a Pharos (Light Conversion Ltd., Lithuania) laser source operating at the wavelength of *λ* = 1.03 *μ*m, pulse duration of *τ* = 200 fs, and a repetition rate of *f* = 200 kHz. For the large aspect ratio waveguides and DOEs (Figs. 2, 4 and 5), we used second harmonic generation (SHG) of the pump beam with a barium borate (BBO) crystal for laser writing, as the shorter 515 nm writing wavelength can be exploited to achieve a higher index change^25^. The 515 nm beam size was magnified by a factor of 3 with a beam expander, to a 1 cm diameter, and then passed through a computer controlled attenuator. An axicon with a 179° apex angle was then used to generate the Bessel beam. A 0.4NA objective was used to focus the beam in order to reduce its axial Bessel zone length (over which it is non-diffractive) and hence control the height of the inscribed structure, as well as improve resolution by reducing the beam width. During writing, a computer controlled translation stage moved the silica substrate precisely in the *x* and *y* directions, while the objective was adjusted in the vertical *z* direction. The overhead camera allowed in-situ visualisation of the writing process and initial quality inspection of the imprinted structures.

The fused silica substrates (UVFS C7980 0F) used in our experiments were manufactured by Altechna Ltd. Initially, we investigated stress distribution around laser written waveguide-like structures. Each waveguide was written with the stage moving in the *x* and *y* directions, to inscribe 100 closely spaced raster scanlines. Each scanning line is written within the 10 mm sample in the *y* direction with a typical scanning line separation of 200 nm in the *x* direction. The edges of the substrate were polished before laser writing to reduce surface scattering. The buried waveguides were written 0.5 mm below the sample surface. A typical pulse energy for Bessel beam writing is approximately 0.5 *μ*J and pulse density is 10^5^ pulses/mm along each scanning line.

For the 10 × 10 *μ*m waveguides in Figs. 3 and 5, the 1030 nm wavelength Gaussian writing beam was used, focused by a 0.4NA objective lens. The pulse energy was 0.3 *μ*J and pulse density 5 × 10^3^ pulses/mm, using 50 scan lines per waveguide.

**Figure 7:**
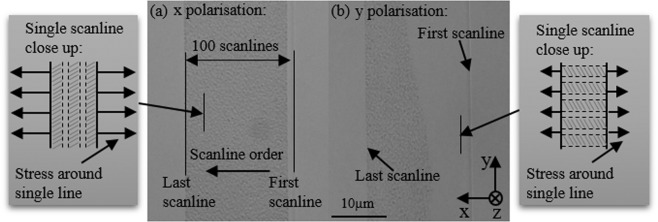
Top view of Bessel beam written waveguide with laser linearly polarised (**a**) perpendicular to waveguide direction (*x* direction) and (**b**) along waveguide direction (*y* direction). Single scanline close up schematics indicate the direction of nano grating and stress around scanline^13^.

The Δ*n* of the structure induced by femtosecond laser depends on the laser parameters (pulse density, pulse energy) within the continuous modification region. Beyond the material damage threshold, we found different damage (nanograting) formation behaviour for the two orthogonal linear polarisation states of the writing beam (see Fig. 7). This is caused by the different stress distribution around laser writing lines. For a *y* polarised laser beam, the generated nanograting planes are perpendicular to the *y* direction (Fig. 7b) and thus has lower stress distributed around a single line than the *x* polarised beam^13^ (Fig. 7a). In order to eliminate the polarisation-dependant damage formation, we use circular polarisation for laser writing, which generates a more isotropic stress distribution^19^.

The stress profiles of the large-aspect ratio waveguides in Figs. 1c, 4 and 5 were obtained via retardance measurements using an optical microscope system with a VariLC liquid crystal device controlled by OpenPolScope software. For further investigation on diffractive optics, we wrote diffractive 50/50 gratings and Fresnel lenses. The grating has a 10 *μ*m period and 1 mm × 1 mm size. The Fresnel lens has a 2 mm diameter and 10 mm focal length. Both the gratings’ first order diffraction efficiency and Fresnel lenses’ focal efficiency were characterised with a 632.5 nm wavelength HeNe laser. The mode profiles of Gaussian beam written 10 × 10 *μ*m waveguides were characterised with a 1310 nm laser diode.

## Conclusion

We demonstrate a stress control technique for femtosecond laser written structures in silica. With this technique, we are able to eliminate the structural asymmetry of waveguide-like structures. A more symmetric stress profile also improves the damage threshold so higher writing powers can be used, giving stronger Δ*n* and phase change. For high aspect-ratio Bessel beam written DOEs, a 36% of diffractive efficiency improvement for a 50/50 grating was achieved compared to the standard multiscan writing technique. A two-layer Fresnel lens with a focal efficiency of 55% was also demonstrated. For low aspect-ratio Gaussian beam written waveguides, a more symmetric mode profile was achieved using the improved scanning technique. In addition to complex diffractive structures, more generally this stress control method could also be adapted for laser machining, microfluidic channel etching and large waveguide fabrication. Furthermore, it can be easily implemented in existing femtosecond writing setups since it does not require any additional hardware.

## References

[1] Pavlov I (2017). Femtosecond laser written waveguides deep inside silicon. Opt. Lett..

[2] Riesen N, Gross S, Love JD, Withford MJ (2014). Femtosecond direct-written integrated mode couplers. Opt. Express.

[3] Beresna M, Gecevičius M, Kazansky PG, Gertus T (2011). Radially polarized optical vortex converter created by femtosecond laser nanostructuring of glass. Appl. Phys. Lett..

[4] Zhang YJ, Zhang GD, Chen CL, Stoian R, Cheng GH (2016). Transmission volume phase holographic gratings in photo-thermo-refractive glass written with femtosecond laser Bessel beams. Opt. Mater. Express.

[5] Zhang, J. *et al*. Eternal 5d data storage by ultrafast laser writing in glass. In *Laser-based Micro-and Nanoprocessing X*, vol. 9736, 97360U (International Society for Optics and Photonics, 2016).

[6] Bellouard Y, Said A, Dugan M, Bado P (2004). Fabrication of high-aspect ratio, micro-fluidic channels and tunnels using femtosecond laser pulses and chemical etching. Opt. Express.

[7] Hnatovsky C (2006). Fabrication of microchannels in glass using focused femtosecond laser radiation and selective chemical etching. Appl. Phys. A.

[8] Champion A, Bellouard Y (2012). Direct volume variation measurements in fused silica specimens exposed to femtosecond laser. Opt. Mater. Express.

[9] Marcinkevičius A (2001). Femtosecond laser-assisted three-dimensional microfabrication in silica. Opt. Lett..

[10] Hnatovsky C (2005). Pulse duration dependence of femtosecond-laser-fabricated nanogratings in fused silica. Appl. Phys. Lett..

[11] Bellouard Y (2016). Stress-state manipulation in fused silica via femtosecond laser irradiation. Opt..

[12] Guo H (2004). The pulse duration dependence of femtosecond laser induced refractive index modulation in fused silica. J. Opt. A: Pure Appl. Opt..

[13] Champion A, Beresna M, Kazansky P, Bellouard Y (2013). Stress distribution around femtosecond laser affected zones: effect of nanogratings orientation. Opt. Express.

[14] Eaton SM, Ng ML, Osellame R, Herman PR (2011). High refractive index contrast in fused silica waveguides by tightly focused, high-repetition rate femtosecond laser. J. Non-Crystalline Solids.

[15] Mikutis M, Kudrius T, Šlekys G, Paipulas D, Juodkazis S (2013). High 90% efficiency bragg gratings formed in fused silica by femtosecond Gauss-Bessel laser beams. Opt. Mater. Express.

[16] Durnin J, Eberly JH, Miceli JJ (1988). Comparison of Bessel and Gaussian beams. Opt. Lett..

[17] Nasu Y, Kohtoku M, Hibino Y (2005). Low-loss waveguides written with a femtosecond laser for flexible interconnection in a planar light-wave circuit. Opt. Lett..

[18] McGloin D, Dholakia K (2005). Bessel beams: diffraction in a new light. Contemp. Phys..

[19] McMillen B, Bellouard Y (2015). On the anisotropy of stress-distribution induced in glasses and crystals by non-ablative femtosecond laser exposure. Opt. Express.

[20] McMillen B, Athanasiou C, Bellouard Y (2016). Femtosecond laser direct-write waveplates based on stress-induced birefringence. Opt. Express.

[21] Chan JW, Huser T, Risbud S, Krol D (2001). Structural changes in fused silica after exposure to focused femtosecond laser pulses. Opt. letters.

[22] Sugiura H, Yamadaya T (1992). Raman scattering in silica glass in the permanent densification region. J. non-crystalline solids.

[23] Bellouard Y, Barthel E, Said A, Dugan M, Bado P (2008). Scanning thermal microscopy and raman analysis of bulk fused silica exposed to low-energy femtosecond laser pulses. Opt. Express.

[24] Agarwal A, Tomozawa M (1997). Correlation of silica glass properties with the infrared spectra. J. Non-Crystalline Solids.

[25] Shah L, Arai AY, Eaton SM, Herman PR (2005). Waveguide writing in fused silica with a femtosecond fiber laser at 522 nm and 1 MHz repetition rate. Opt. Express.

